# In-hospital antimicrobial use and multidrug-resistant infections among COVID-19 patients in Northern Malaysia (COVAM): a multicentre study

**DOI:** 10.1080/20523211.2026.2728836

**Published:** 2026-09-28

**Authors:** Kah Shuen Thong, Chii-Chii Chew, Meng Fei Cheah, E-Li Leong, Chee Tao Chang, Jason Choong Yin Lee, Philip Rajan Devesahayam, Hong Bee Ker, Jeyaseelan P. Nachiappan, Zaiton Kamarruddin, Lay Ming Ding

**Affiliations:** a Pharmacy department, Hospital Raja Permaisuri Bainun, Ministry of Health, Ipoh, Malaysia; b Pharmacy Department, Jelapang Health Clinic, Kinta Health District, Perak, Malaysia; c Clinical Research Centre, Hospital Raja Permaisuri Bainun, Institute for Clinical Research, National Institute of Health, Ministry of Health Malaysia, Ipoh, Malaysia; d Clinical Research Centre, Hospital Raja Permaisuri Bainun, Ministry of Health, Ipoh, Malaysia; e Perak Pharmaceutical Services Division, Ministry of Health, Ipoh, Malaysia; f Infectious Disease Unit, Medical Department, Hospital Raja Permaisuri Bainun, Ministry of Health, Ipoh, Malaysia; g Faculty of Medicine, Universiti Kuala Lumpur, Royal College of Medicine Perak, Ipoh, Malaysia; h Perak State Health Department, Ministry of Health, Ipoh, Malaysia

**Keywords:** COVID-19, anti-infective agent, anti-bacterial agent, antibiotic, antifungal agent, multidrug-resistant organisms

## Abstract

**Background:**

During the COVID-19 pandemic, empirical antimicrobials were frequently prescribed despite low documented rates of bacterial and fungal co-infections. While early pandemic data exist, evidence evaluating antimicrobial consumption and multidrug-resistant organism (MDRO) dynamics during subsequent pandemic surges remains limited. This study evaluated antimicrobial receipt, associated factors, and the prevalence of MDROs among hospitalised COVID-19 patients in Northern Malaysia.

**Methods:**

This multicentre retrospective observational cohort study included adults (≥18 years) with confirmed COVID-19 admitted to ten government hospitals in Northern Malaysia between February 2020 and December 2021. Among 19,116 admissions, 480 cases were sampled, yielding 476 eligible patients. Study outcomes included antimicrobial receipt and MDRO prevalence. Monthly antimicrobial days of therapy (DOT) per 1,000 patient-days were compared with state-level COVID-19 hospital admissions. Predictors were analysed using a Poisson Generalized Linear Mixed Model.

**Results:**

Antibiotics were prescribed to 167 patients (35.1%), accounting for 381 prescriptions, predominantly β-lactam/β-lactamase inhibitors (54.1%). Most were empirical (92.9%), largely among non-ICU patients (72.2%) with pneumonia (82.9%). Antifungals were used in 13 patients (2.7%; 15 prescriptions), mainly empirical echinocandins (60.0%), and predominantly in ICU settings (66.7%). MDROs were detected in 8.4% (10/119) of patients with cultured specimens, representing patient-level MDRO prevalence. A significant positive correlation was observed between DOT and admissions (r = 0.701, *p* < 0.001, 95% CI [0.37–0.87]). Severe/critical COVID-19 stage, diabetes mellitus, chronic obstructive pulmonary disease and chronic kidney disease were consistently associated with antimicrobial prescription. Longer hospital stays, elevated neutrophil and C-reactive protein levels, smoking, and pre-hospital steroid/immunosuppressant use were additional predictors.

**Conclusion:**

Antimicrobial use was predominantly empirical and influenced by disease severity and admission surges. Although MDRO detection remained low, severity-driven prescribing highlights the risk of unnecessary antimicrobial exposure during pandemic waves. These findings inform outbreak-responsive antimicrobial stewardship strategies and support antimicrobial utilisation surveillance to guide resource planning during future public health emergencies.

## Introduction

Although early uncertainty prompted widespread empirical antimicrobial use, evidence shows low bacterial co-infection rates in COVID-19 patients (3.8–7.4%, 8.0% in Intensive Care Unit [ICU]) alongside persistently high antibiotic prescribing (50–75%) (Langford et al., 2023; Weiner-Lastinger et al., 2022). In parallel, COVID-19 was also associated with a higher burden of invasive fungal infections than non-COVID-19 populations, with incidence ratios of 4.64 overall and 2.52 in ICUs. Notably, fungal co-infections reported in up to 28.2% of critically ill patients, driving increased empirical antifungal consumption and exacerbating antimicrobial stewardship concerns (Seaton et al., 2020).

Before COVID-19, antimicrobial stewardship (AMS) programmes reduced unnecessary antibiotic prescribing through diagnostic protocols and biomarkers such as procalcitonin (Borek et al., 2021; Drewett et al., 2022). In contrast, during the pandemic, antimicrobial prescribing shifted towards empirical use of broad-spectrum antibiotics, while use of narrower-spectrum agents declined. These prescribing patterns were largely driven by disease severity, ICU admission, and elevated inflammatory markers, potentially undermining antimicrobial stewardship efforts (Rozman et al., 2025; Ruzsa et al., 2024).

Studies suggest that the COVID-19 pandemic was associated with increased healthcare-associated infections (HAIs) and multidrug-resistant organisms (MDROs), particularly in regions experiencing a high COVID-19 burden. A higher rate of carbapenem-resistant *Acinetobacter baumannii*, antifungal-resistant *Candida*, extended-spectrum β-lactamase (ESBL)-producing Enterobacterales, and vancomycin-resistant enterococci (VRE) was reported. In contrast, trends for carbapenem-resistant Enterobacterales (CRE), multidrug-resistant (MDR) *Pseudomonas aeruginosa*, and methicillin-resistant *Staphylococcus aureus* (MRSA) remained relatively stable (Abdelaziz Abdelmoneim et al., 2024; Witt et al., 2022). However, these trends were not universal, as some specialised healthcare settings reported no significant increase in HAIs, suggesting that local epidemiology and infection prevention practices may have influenced resistance patterns (Petrone et al., 2024). Meanwhile, high baseline MDRO prevalence was observed in long-term care facilities, emphasising the role of vulnerable populations as reservoirs for resistant organisms (Ma et al., 2025).

Similar concerns have been reported in Malaysia. National surveillance has documented substantial resistance among clinically important pathogens, including ESBL-producing *Klebsiella pneumoniae*, carbapenem-resistant organisms, MDR *A. baumannii*, and MRSA (Ramli et al., 2023). Nevertheless, antimicrobial use during the early pandemic remained relatively low, with few microbiologically confirmed bacterial or fungal infections, and reduced antibiotic consumption was also observed in primary care (Lim et al., 2023; Mohamad et al., 2022; Ramli et al., 2023). Because these findings primarily represent the early phase of the pandemic, evidence describing antimicrobial prescribing and MDRO emergence during subsequent waves remains limited (Supramanian et al., 2021).

To address this knowledge gap, this study examined antimicrobial use and MDROs among hospitalised COVID-19 patients in Northern Malaysia from February 2020 to December 2021, encompassing the second and third pandemic waves (Tan et al., 2021). This period was characterised by greater disease burden, prolonged hospitalisation, increased empirical antimicrobial prescribing, and disruptions to infection prevention practices, all of which may have contributed to the emergence and transmission of MDROs (Dabaja-Younis et al., 2024). Given the growing global concern regarding antimicrobial resistance associated with COVID-19, local evidence is needed to better understand prescribing practices and resistance patterns in real-world settings (Bhargava et al., 2021). Therefore, this study evaluated in-hospital antimicrobial use, identified clinical predictors of antibiotic and antifungal prescribing, and determined the prevalence of MDR infections among hospitalised COVID-19 patients in Northern Malaysia during the first two years of the pandemic to inform antimicrobial stewardship and public health strategies.

## Methods

### Study designs

This multicentre retrospective observational cohort study reviewed COVID-19 patient records from ten government hospitals in northern Malaysia. Ethical approval was obtained from the Malaysian Medical Research and Ethics Committee (MREC) with the NMRR registration number ID-21-02162-VV3 (IIR). As this study used retrospective data, informed consent was waived.

### Population

#### Inclusion criteria

The medical records of all patients aged ≥18 years with confirmed COVID-19 admitted between 1 February 2020 and 31 December 2021 were included. A confirmed COVID-19 case was defined as either a positive Rapid Test Kit Antigen (RTK-Ag) result in high-prevalence areas (≥10% COVID-19 prevalence) or a positive molecular test (RT-PCR or rapid molecular test), regardless of patient outcome (Ministry of Health Malaysia, 2023a).

#### Exclusion criteria

Patients who only received care in temporary holding areas (such as COVID Assessment Centres) or the Emergency Department without subsequent hospital admission were excluded.

### Sample size and sampling method

Antibiotic use among COVID-19 patients ranged from 34.2% to 71.0% across countries (Beović et al., 2020; Singh et al., 2021). The required sample size for estimating a single proportion was calculated using the standard formula:

$$n=\frac{Z^{2}P(1-P)}{d^{2}}$$

where *Z* = 1.96 (95% confidence level), *P* = 0.50 (conservative expected prevalence to maximise sample size), and *d* = 0.05 (precision). This yielded a minimum sample size of 384 participants. Although G*Power 3.1.9.4 was used to verify calculations, the primary estimation was based on the single-proportion formula.

For antifungals (0.1%–9.8% prevalence) (Seaton et al., 2020; Singh et al., 2021), the minimum sample size was calculated using the single proportion formula where *Z* = 1.96 for 95% confidence, *P* = 0.1%–9.8%, and *d* = 0.05. The calculation yielded a range of 2–136 patients; however, a larger sample of 384 was adopted to ensure sufficient study power.

Allowing 20% for missing data, the final target was 480 was established a priori. Sampling was conducted using proportionate stratified simple random sampling, where sampling fractions were calculated based on the bed capacity of each participating hospital. Eligible patients were randomly selected using Sealed Envelope® v1.21.0. Individual data for non-selected patients were not extracted per the protocol, as data extraction was restricted solely to the randomly selected sample.

Model adequacy for the multivariable Poisson Generalized Linear Mixed Model (GLMM) analysis was supported by an events-per-variable of 11.1 (167 antibiotic-prescribing events and 15 predictors), exceeding the commonly recommended minimum of 10 and considered sufficient for stable estimation (Peduzzi et al., 1996).

### Instrument

A standardised case report form (CRF), as shown in Supplemental Appendix 1, was used to capture demographics, comorbidities, admission details (ICU/non-ICU), World Health Organization COVID-19 severity at admission and worst progression, vaccination status, pre-hospital immunosuppressive therapy, laboratory results (complete blood count, liver/renal function, C-reactive protein [CRP]), antimicrobial therapy (initiation, adjustments, duration), immunomodulator use, oxygen requirements, culture results, complications, and outcomes (discharge or death) (World Health Organization, 2023). MDROs were defined as microorganisms resistant to ≥1 agent in ≥3 antimicrobial classes (Magiorakos et al., 2012); these included *Stenotrophomonas maltophilia*, MRSA, carbapenem-resistant *K. pneumoniae* (CRE), *A. baumannii* (MDR), *Acinetobacter* sp. (MDR), and ESBL-producing organisms. This classification was used by the microbiology laboratories at our study sites. MDRO status was recorded directly from the culture and sensitivity reports in the patients’ case records without additional interpretation by the data collector. Following content and face validity review of the CRF by investigators and infectious disease specialists, data were retrospectively collected from paper and electronic medical records.

### Training and data collection

Data collectors were experienced ward pharmacists trained on all CRF variables, with ongoing support from the coordinating team. Inter-rater reliability was not assessed, as data collectors transcribed only documented information. Data extraction was based on predefined variables with explicit operational definitions. Data collectors were instructed to extract information directly from the medical records without requiring interpretation. A random 10% sample of records underwent independent verification against the source case records to assess data accuracy. Although it is often assumed that reviewing a higher proportion of records would enhance data quality, this assumption has not been empirically validated. Previous guidance recommends that a random sample of 5–10% of participant records is sufficient for auditing with source documents (Houston et al., 2018).

### Missing data

Inferential analysis was performed using a GLMM with Poisson regression. Missing data, assumed missing at random (MAR), were addressed using multiple imputation by chained equations in RStudio (v2025.09.0 + 387). To evaluate the missing data mechanism, Little’s MCAR test was conducted and was statistically significant (*χ²* = 22,514,176,172.87, df = 2,829, *p* < 0.001), indicating that the data were not missing completely at random (MCAR). Therefore, multiple imputation under the MAR assumption was considered an appropriate approach (Austin et al., 2021). Admission inflammatory markers (neutrophil count, lymphocyte count, CRP) were conditionally imputed based on antimicrobial initiation date, alongside demographic, clinical, and laboratory predictors informed by prior studies (Gan et al., 2024; Langford et al., 2020; Mohamad et al., 2022). Five datasets were generated using predictive mean matching over ten iterations and pooled for analysis. Descriptive checks showed no difference between original and imputed means (Supplemental Appendix 2).

The stability of multiple imputation was assessed using three imputations across five random seeds (2024, 12345, 24680, 13579, 54321). Variability of pooled estimates was quantified by the coefficient of variation (CV = Standard deviation [SD] / mean × 100), with CV <5% indicating excellent stability (Lu, 2017). CRP, the variable with the highest missingness, showed good stability (CV = 4.49%), while lymphocytes had moderate variability (CV = 29.8%) but a modest absolute SD (0.80 units) (Supplemental Appendix 3). These results implied that five imputations were sufficient, consistent with recommendations for high relative efficiency with modest missing information (Rubin, 1987; White et al., 2011).

### Data analysis

Data analysis was conducted using RStudio (version 2025.09.2 + 418) with R version 4.4.1 on Windows 11 Pro. Primary outcomes were the proportion of patients with antimicrobials receipt and the number of antimicrobial prescriptions (antibiotics, antifungals, or both) among hospitalised COVID-19 patients. Monthly antimicrobial therapy days per 1,000 patient-days were compared with statewide COVID-19 admission rates during the study period. Descriptive analyses assessed antimicrobial prescription duration, culture and sensitivity results, microorganism types, and the incidence of MDROs.

A Poisson GLMM with a log link examined factors associated with the total number of antimicrobials prescribed among hospitalised COVID-19 patients, using a staged covariate modelling approach guided by literature and univariate screening (*p* < 0.25) (Bursac et al., 2008).

The minimal model included age, sex, comorbidities (diabetes mellitus [DM], hypertension, chronic obstructive pulmonary disease [COPD], chronic kidney disease [CKD]), and COVID-19 severity (Yang et al., 2024), with additional adjustments for laboratory parameters (neutrophil count, lymphocyte count, and CRP), and hospital stay for the primary model (Mohamad et al., 2022). The extended model additionally adjusted for controversial factors in COVID-19 prevalence (e.g. smoking status) (Salehi et al., 2023), policy-influenced variables (e.g. COVID-19 vaccination) (Ministry of Health Malaysia, 2025), and the uncertain effects of pre-hospitalisation immunosuppressant use, including corticosteroids, on antimicrobial use (Davelaar et al., 2023; Zhuang et al., 2024).

Poisson GLMMs were fitted using the glmer function with random intercepts for study site to account for clustering. Pooled incidence rate ratios (IRRs) and 95% confidence intervals (CIs) were estimated using Zeger’s approximation, with statistical significance defined as *p* < 0.05. Model validity was evaluated by testing overdispersion via Pearson $\chi^{2}$ goodness-of-fit tests, where a dispersion ratio > 1.00 (*p* < 0.05) indicated overdispersion. The model's discriminative ability to identify patients with at least one antimicrobial receipt was assessed using the area under the receiver operating characteristic curve (AUC) with 95% CIs.

## Results

Of 480 randomly sampled records, 476 COVID-19 cases were included (Figure 1). The median age was 53 years (Interquartile range [IQR]: 30.0); most patients were female (54.4%), Malay (66.0%), and had at least one comorbidity (70.6%). Factors associated with antimicrobial receipt included age ≥60 years, smoker status, ethnicity of other background (e.g. Orang Asli), and presence of DM, whereas receiving at least one dose of a COVID-19 vaccine or having an unknown vaccination status was associated with lower odds of antimicrobial receipt relative to unvaccinated patients (Table 1).

**Figure 1.**
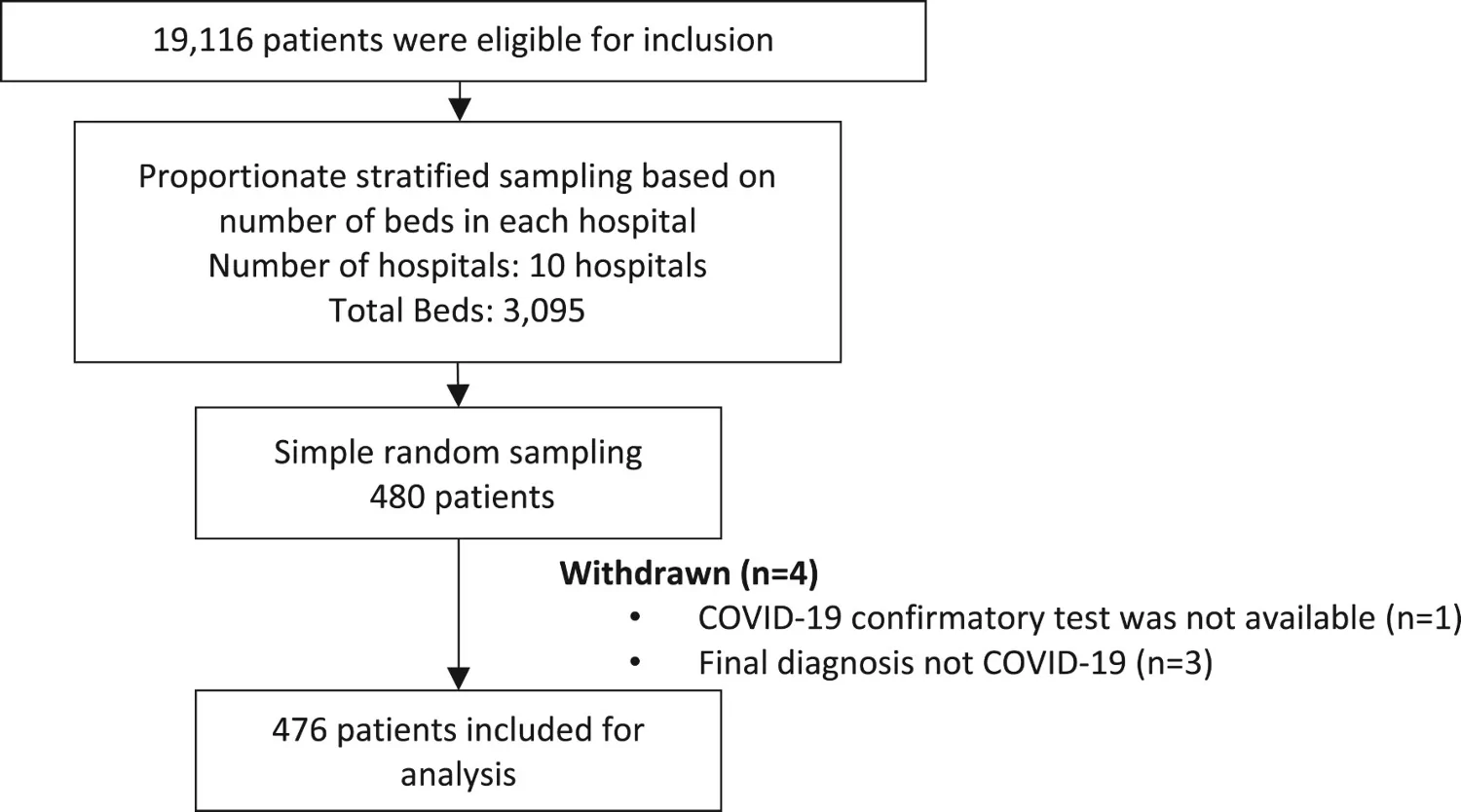
Sampling process and patient inclusion for the study (refer to Supplemental Appendix 4 for the number of eligible patients and their allocation by hospital).

**Table 1.** Demographic characteristics of hospitalised COVID-19 patients.

Variables	Total, *n* (%) (*n* = 476)	Received antimicrobial (*n* = 167)	Unadj. *p*-value	Crude OR (95% CI)	Adj. OR (95% CI)
Age^a^			<0.001		
18–59 years old	291 (61.1)	83 (28.5)		Ref.	
≥60 years old	185 (38.9)	84 (45.4)		2.08 (1.42–3.07)	1.70 (1.03–2.80)
Gender^a^			0.301		
Female	259 (54.4)	85 (32.8)		Ref.	
Male	217 (45.6)	82 (37.8)		1.24 (0.85–1.81)	1.10 (0.69–1.74)
Smoking status^a^			0.015		
Non-smoker	137 (28.8)	51 (37.2)		Ref.	
Smoker	43 (9.0)	23 (53.5)		1.94 (0.97–3.90)	2.22 (1.00–4.98)
Unknown	296 (62.2)	93 (31.4)		0.77 (0.51–1.18)	0.92 (0.55–1.48)
Ethnicity^a^					
Malay	314 (66.0)	105 (33.4)	0.564	Ref.	
Chinese	73 (15.3)	30 (41.1)		1.39 (0.82–2.33)	1.08 (0.59–1.97)
Indian	57 (12.0)	19 (33.3)		1.00 (0.54–1.79)	0.85 (0.43–1.60)
Other (e.g. orang asli)	32 (6.7)	13 (40.6)		1.36 (0.63–2.84)	2.26 (1.00–5.03)
Comorbidities^a^					
Diabetic mellitus	147 (30.9)	65 (44.2)	0.007	1.76 (1.18–2.63)	1.66 (1.00–2.75)
Hypertension	203 (42.6)	83 (40.9)	0.028	1.56 (1.06–2.28)	0.85 (0.51–1.41)
Dyslipidaemia	78 (16.4)	36 (46.2)	0.035	1.75 (1.07–2.86)	1.38 (0.77–2.45)
Obesity (BMI > 30 kg/m^2^)	59 (12.4)	18 (30.5)	0.521	0.79 (0.43–1.40)	1.04 (0.53–2.01)
Chronic pulmonary disease	40 (8.4)	18 (45.0)	0.230	1.58 (0.81–3.03)	1.70 (0.83–3.48)
Chronic kidney disease	29 (6.1)	15 (51.7)	0.082	2.08 (0.97–4.47)	1.73 (0.76–3.97)
Rheumatological disorder	16 (3.4)	7 (43.8)	0.637	1.46 (0.51–3.99)	1.34 (0.43–3.99)
Neurological condition	14 (2.9)	9 (64.3)	0.041	3.46 (1.18–11.4)	2.80 (0.89–9.80)
Others (e.g. Immune deficiencies)	51 (10.7)	24 (47.1)	0.082	1.75 (0.97–3.15)	1.85 (0.68–5.65)
Prior continuous treatment immunosuppressant^a,b^	18 (3.8)	11 (61.1)	0.035	3.04 (1.17–8.41)	1.90 (0.68–5.65)
COVID-19 vaccination^a^			0.005		
Unvaccinated	155 (32.6)	64 (41.3)		Ref.	
≥ one dose	181 (38.0)	69 (38.1)		0.88 (0.56–1.36)	0.72 (0.44–1.17)
Unknown	140 (29.4)	34 (24.3)		0.46 (0.27–0.75)	0.44 (0.26–0.76)

Abbreviations: BMI: Body mass index; OR: Odd ratio; CI: Confidence Interval; Unadj: unadjusted; Adj: Adjusted.

^a^ Analysed with the Pearson’s χ^2^-test, ^b^Including corticosteroid. Adj. OR analysed with multivariate logistics regression.

Chronic pulmonary disease including asthma and chronic obstructive pulmonary disease, Rheumatological disorder including Systemic Lupus Erythematosus and gout.

Antimicrobials were given to 35.1% (n = 167) of patients, totalling 381 prescriptions. β-lactam/β-lactamase inhibitors made up 54.1%, mainly for pneumonia (82.9%), with a median duration of 3 days (IQR: 4.0). Most use (64.1%) occurred in severe or critical COVID-19 cases.

Systemic antifungals were given to 13 patients (2.7%), accounting for 15 prescriptions – 12 empirical and three targeted. Fungal cultures were performed in six cases; three yielded positive results that guided targeted therapy, while three were negative (one obtained postmortem and two after completion of empirical treatment). Seven patients on empirical therapy without cultures died. Treatment duration ranged between 7 and 14 days.

Among the 167 patients with antimicrobials receipt, 119 (71.3%) underwent microbiological culture testing, yielding 335 cultures. Of these, 92 (27.5%) demonstrated microbial growth. MDROs were identified in 15 of these 92 positive cultures (16.3%), with *S. maltophilia* being the predominant organism (53.3%). At the patient level, MDROs were isolated from 10 of the 119 tested patients (8.4%), corresponding to 6.0% of all antimicrobial recipients (10/167) and 2.1% of the overall study cohort (10/476). Patients with MDRO isolation (*n* = 10) experienced significantly longer hospital stays [median: 21 days (IQR: 10.3) vs. 7 days (IQR: 4.0); *p* < 0.001] and higher mortality (50.0% vs. 7.3%; *p* = 0.001) compared to those without MDROs (Table 2).

**Table 2.** Antimicrobial usage and culture sensitivity findings.

Variables		*n* (%)	*p*-value
Antibiotic Use (*n* = 476)	Patients prescribed ≥1 antibiotic	167 (35.1)	
COVID-19 stage upon initiation (*n* = 167)	Mild to moderate	60 (35.9)	<0.001
	Severe to critically ill	107 (64.1)	
Classes of antibiotics prescribed (*n* = 381)	β-lactam/β-lactamase inhibitors	206 (54.1)	
	Macrolides	76 (19.9)	
	1st/2nd-gen cephalosporins	23 (6.0)	
	3rd/4th-gen cephalosporins	34 (8.9)	
	Carbapenems	25 (6.6)	
	Others^a^	17 (4.5)	
Diagnosis (*n* = 381)	Pneumonia	316 (82.9)	
	Bacteraemia	34 (8.9)	
	Others^b^	31 (8.1)	
Location (*n* = 381)	Non-ICU	275 (72.2)	
	ICU	106 (27.8)	
Indication (*n* = 381)	Empirical	354 (92.9)	
	Definitive	22 (5.8)	
	Prophylaxis	5 (1.3)	
Antifungal use (*n* = 476)	Patients prescribed antifungals	13 (2.7)	
COVID-19 stage upon initiation (*n* = 13)	Severe to critically ill	13 (100.0)	<0.001^c^
Total antifungal prescribed (*n* = 15)	Fluconazole	6 (40.0)	
	Micafungin	5 (33.3)	
	Anidulafungin	3 (20.0)	
	Caspofungin	1 (6.7)	
Diagnosis (*n* = 15)	Fungaemia	9 (60.0)	
	Pneumonia	3 (20.0)	
	UTI	2 (13.3)	
	Others	1 (6.7)	
Location (*n** *= 15)	ICU	10 (66.7)	
	Non-ICU	5 (33.3)	
Indication (*n* = 15)	Empirical	12 (80.0)	
	Targeted	3 (20.0)	
	Fungaemia	9 (60.0)	
Microbiology testing	Patients underwent culture tests	119 (71.3)	
	Patients with MDRO isolates	10 (8.4)	
Culture Site (*n* = 335)	Blood	225 (67.2)	
	Urine	52 (15.5)	
	Tracheal	39 (11.6)	
	Sputum	14 (4.2)	
	Stool	5 (1.5)	
Culture results (*n* = 335)	Positive cultures	92 (27.5%)	
Organism Type (*n* = 92)	Gram-negative	38 (41.3)	
	Gram-positive	26 (28.3)	
	Fungi	11 (12.0)	
	Others	17 (18.5)	
MDRO Identified (*n* = 15)	MDRO isolates (n = 92)	15 (16.3)	
	*Stenotrophomonas maltophilia*	8 (53.3)	
	MRSA	2 (13.3)	
	*Klebsiella pneumoniae* (CRE)	2 (13.3)	
	*Acinetobacter baumannii* (MDR)	2 (13.3)	
	*Acinetobacter* sp. (MDR)	1 (6.7)	

Abbreviations: ICU: Intensive Care Unit; UTI: Urinary tract infection; MDRO, multidrug-resistant organism; MRSA, methicillin-resistant *Staphylococcus aureus*; CRE, carbapenem-resistant Enterobacterale; MDR, multidrug-resistant. *P*-value analysed using Chi-square test.

^a^ Others (antibiotics): lincosamides, fluoroquinolones, nitroimidazoles, glycopeptides, sulfonamides, polymyxins.

^b^ Others (diagnoses): UTI, skin & soft tissue infections, and miscellaneous.

^c^ *P*-value analysed using Fisher’s exact test.

**Prophylaxis:** Antibiotics given to prevent infection in patients at risk, per clinical guidelines.

**Pre-emptive:** Antifungal therapy started based on diagnostic findings (e.g. β-D-glucan, galactomannan, imaging) suggesting infection.

**Empirical:** Antimicrobials initiated on clinical suspicion of infection without microbiological confirmation.

**Targeted/Definitive:** Therapy guided by pathogen identification and susceptibility results.

The cohort included a substantial proportion of severe/critical COVID-19 cases: 31.5% at admission and 38.9% during hospitalisation. Despite low ICU admission (2.1%), it was significantly associated with antimicrobial use (*p* < 0.001). Most laboratory values upon hospital admission were normal (52.3–91.9%). Clinical management measures were common: 42.0% required oxygen support, of whom 61.5% received antimicrobials (*p* < 0.001) (Supplemental Appendix 5).

Immunomodulators were used in 41.0% of patients, with 65.1% also having antimicrobials receipt (*p* < 0.001). Patients who received antimicrobial therapy had longer hospital stays (median: 8 days, IQR: 9.0) compared to those who did not receive antimicrobials (median: 7 days, IQR: 5.0; *p* < 0.001). Clinical outcomes were largely favourable, with 86.3% discharged without complications.

Significant differences in antimicrobial distribution were observed between COVID-19 severity groups, most notably within β-lactam/inhibitor combinations, where amoxicillin/clavulanate predominated in mild/moderate cases (58.8%) while piperacillin/tazobactam was used almost exclusively in severe/critical patients (90.7%). Significant associations with severity were also identified for macrolides, carbapenems, and cephalosporins. Conversely, usage of antifungals and glycopeptides did not differ significantly between groups ( Supplemental Appendix 6 ).

Figure 2 presents COVID-19 admissions (n = 8,706) and antimicrobial use, expressed as days of therapy (DOT) per 1,000 patient-days, during Malaysia’s second and third pandemic waves (Ministry of Health Malaysia, 2022). Admissions peaked in January, May, and August–September 2021. In contrast, antimicrobial use increased more consistently over time, rising steadily from January to November, with only a transient decline observed in April. From August to November 2021, DOT generally tracked admission trends, although temporal patterns were not fully aligned.

**Figure 2.**
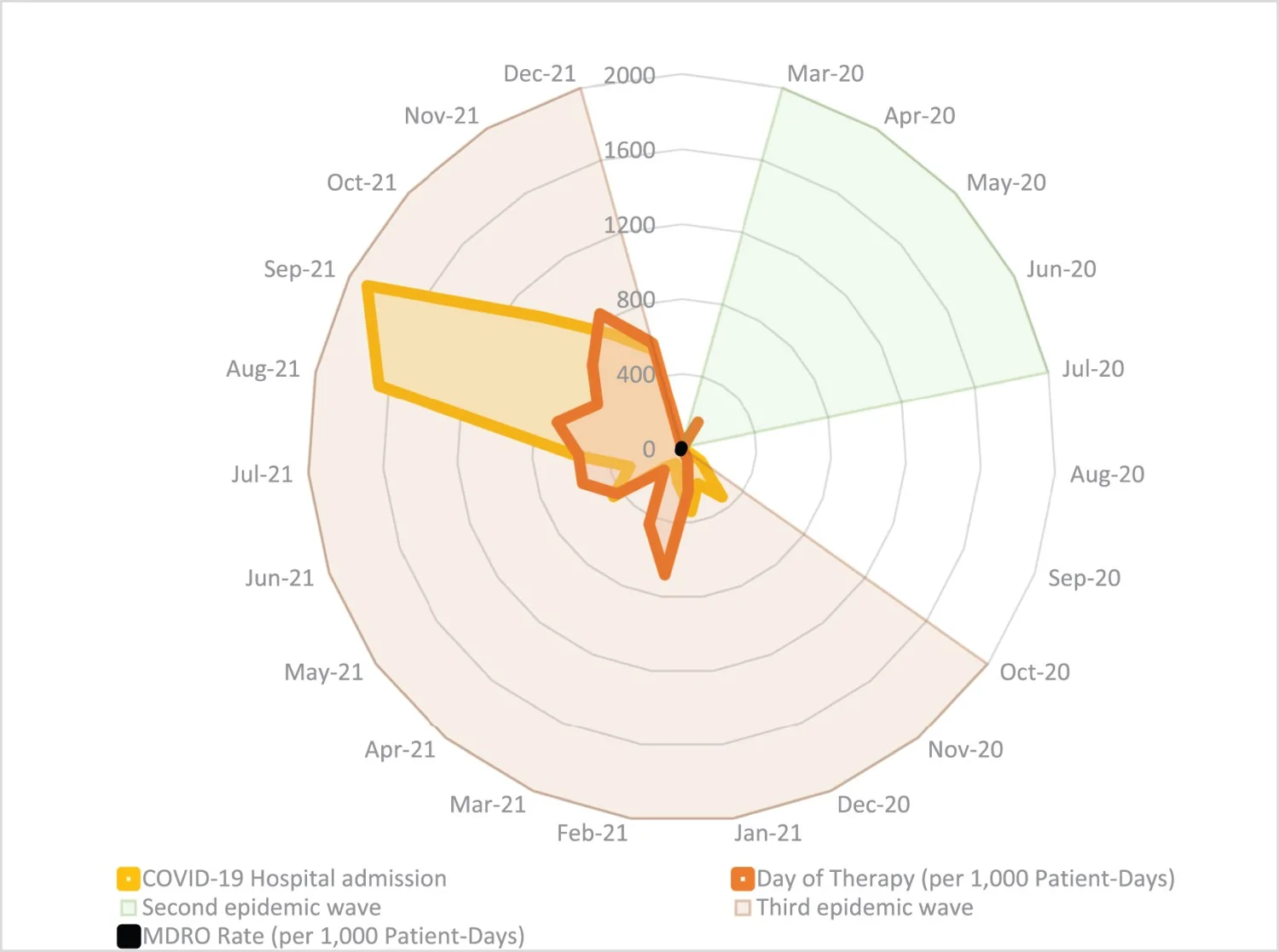
Temporal relationship between COVID-19 hospital admissions, antimicrobial use (days of therapy [DOT] per 1,000 patient-days), and MDRO incidence (per 1,000 patient-days) during the second and third pandemic waves in Malaysia.

Two notable divergences were observed. In February 2021, antimicrobial use was disproportionately high (682.2 DOT per 1,000 patient-days) despite relatively low admissions (*n* = 179). Conversely, while admissions reached their highest level in September 2021 (*n* = 1,895), the peak in antimicrobial use occurred later in November 2021 (844.0 DOT per 1,000 patient-days), reflecting an observed temporal association with an approximate two-month lag between peak case burden and maximal antimicrobial utilisation. Overall, Pearson’s correlation analysis demonstrated a significant positive association between admissions and antimicrobial use (r = 0.701, *p* < 0.001, 95% CI [0.37, 0.87]), suggesting that higher COVID-19 case burdens were generally associated with increased antimicrobial prescribing.

MDRO values appear visually minimal in the overall radar chart (Figure 2, black markers). The incidence density rates remained at 0.00 per 1,000 patient-days throughout 2020. In 2021, rates increased, with an isolated spike in March (16.39 per 1,000 patient-days), followed by a smaller peak in June 2021 (5.35 per 1,000 patient-days). From August to December 2021, incidence density rates remained persistently elevated (2.32–5.75 per 1,000 patient-days), coinciding with peak COVID-19 hospital admissions and antimicrobial consumption.

In the Poisson GLMM minimal model (AIC = 79,156.04; BIC = 79,240.87; dispersion = 1.59, *p* < 0.001; AUC = 0.803, 95% CI: 0.798–0.808), critical/severe COVID-19 stage was associated with higher antimicrobial use (IRR = 3.52, 95% CI: 3.42–3.61), along with DM (IRR = 1.14, 95% CI: 1.11–1.17), hypertension (IRR = 1.11, 95% CI: 1.08–1.15), COPD (IRR = 1.30, 95% CI: 1.19–1.42), and CKD (IRR = 1.53, 95% CI: 1.47–1.59). Male gender was modestly associated with higher antimicrobial use (IRR = 1.06, 95% CI: 1.03–1.08), and age showed a small but significant effect (IRR = 1.001, 95% CI: 1.000–1.002).

The primary model (AIC = 10,399.24; BIC = 10,492.9; dispersion ratio = 0.96, *p* = 0.949; AUC = 0.883, 95% CI: 0.872–0.893) showed that severe/critical COVID-19 stage was associated with higher antimicrobial use (IRR = 1.77, 95% CI: 1.65–1.90). DM (IRR = 1.37, 95% CI: 1.28–1.47), COPD (IRR = 1.25, 95% CI: 1.02–1.54), CKD (IRR = 1.18, 95% CI: 1.09–1.29), and longer hospital stay (IRR = 1.05 per day, 95% CI: 1.05–1.06) were also associated with increased antimicrobial use, while hypertension showed a modest inverse association (IRR = 0.93, 95% CI: 0.87–0.99). Higher neutrophil count and CRP were positively associated with antimicrobial use, whereas lymphocyte count showed a small inverse effect. Age, gender, and dyslipidaemia were not significant predictors.

The extended model (AIC = 10,393.52; BIC = 10,512.16; dispersion ratio = 0.97, *p* = 0.924; AUC = 0.884, 95% CI: 0.874–0.894) showed that severe/critical COVID-19 stage (IRR = 1.65, 95% CI: 1.53–1.79), DM (IRR = 1.38, 95% CI: 1.29–1.48), COPD (IRR = 1.26, 95% CI: 1.02–1.56), CKD (IRR = 1.23, 95% CI: 1.12–1.35), smoking (IRR = 1.10, 95% CI: 1.02–1.19), and pre-hospital steroid or immunosuppressant use (IRR = 1.10, 95% CI: 1.01–1.19) were associated with higher antimicrobial use. Longer hospital stays (IRR = 1.05 per day, 95% CI: 1.05–1.05) was also a predictor, whereas hypertension (IRR = 0.92, 95% CI: 0.86–0.98) showed inverse associations. The three inflammatory markers in this model demonstrated patterns consistent with those observed in the primary model.

Across the three models, severe/critical COVID-19 stage, DM, hypertension, COPD, and CKD remained consistent predictors of antimicrobial use. Longer hospital stays and deranged inflammatory markers showed consistent associations in the primary and extended models, whereas dyslipidaemia remained non-significant across both models (Figure 3) (Supplemental Appendix 7).

**Figure 3.**
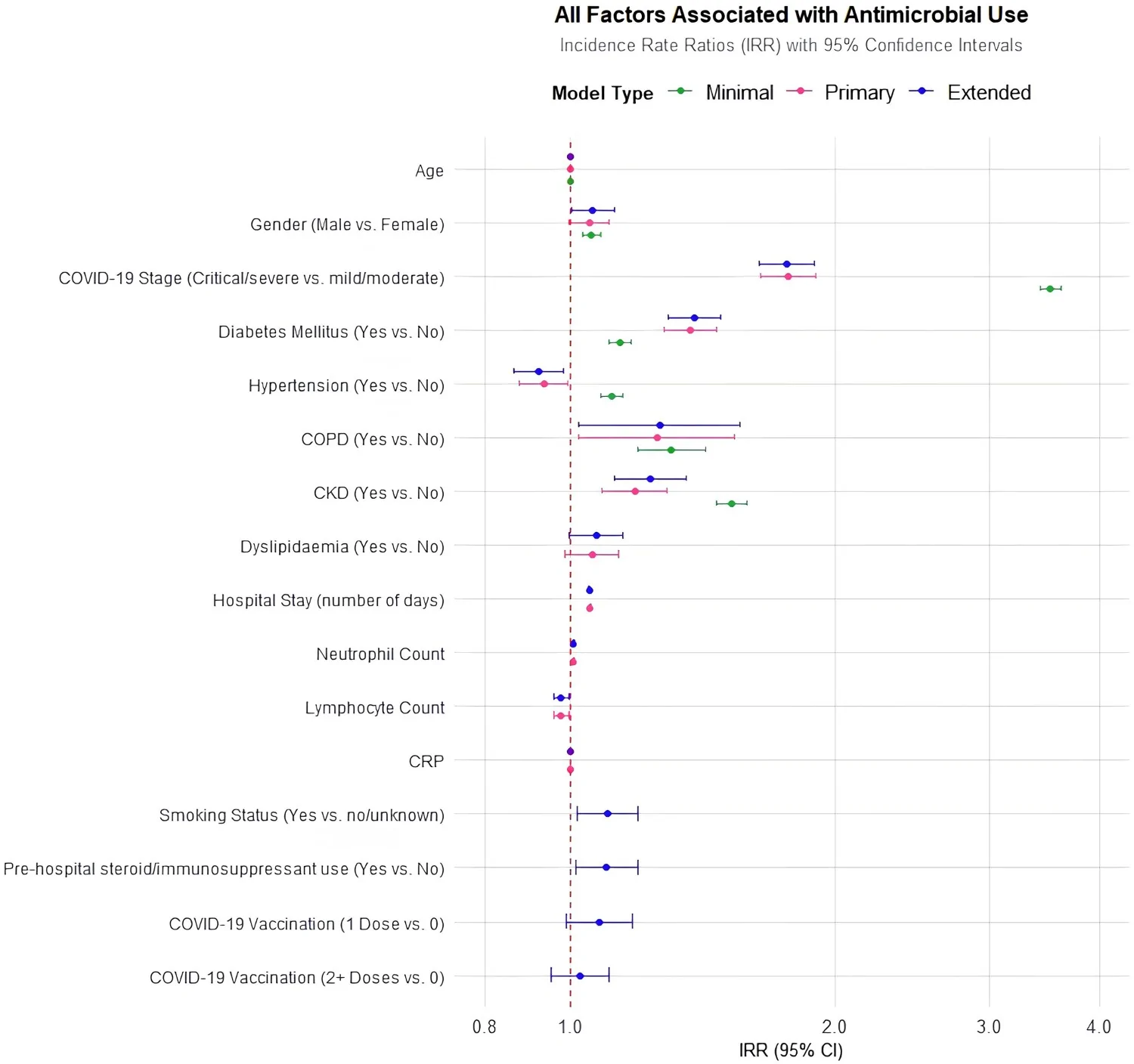
Factors associated with increased antimicrobial use in hospitalised COVID-19 patients. Abbreviations: IRR, Incidence Rate Ratio; 95% CI, 95% Confidence interval; COPD, Chronic obstructive pulmonary disease; CKD, Chronic kidney disease; CRP: C-reactive protein.

The Poisson GLMM minimal model showed overdispersion (dispersion ratio = 1.590). This model was constructed based on variables identified from the literature and was included mainly for comparison of variable effects. As the minimal model was not intended to represent the final analytical model, negative binomial regression was not applied to address the observed overdispersion. In contrast, the primary and extended models demonstrated improved dispersion diagnostics, with dispersion ratios of 0.96 and 0.97, respectively, indicating that the Poisson variance assumption was reasonably satisfied.

## Discussion

This study showed that in-hospital antimicrobial use among COVID-19 patients in Northern Malaysia (35.1%) was within the lower range of global estimates (32.9–85.6%) but exceeded early-pandemic Malaysian reports (6.9%) (Ramli et al., 2023; Sili et al., 2024). The higher use compared with early-pandemic data likely reflects changes in clinical practice over time, increased disease burden during subsequent pandemic waves, and greater exposure to prolonged hospitalisation and complications. Most prescriptions targeted pneumonia and predominantly involved β-lactam/β-lactamase inhibitors and macrolides, consistent with recommended empirical management of suspected bacterial co-infections in COVID-19 patients (Ministry of Health Malaysia, 2023b; Papst et al., 2022).

The observed antimicrobial use highlights substantial variation across Malaysian healthcare settings. In-hospital antibiotic use in our study (35.1%) exceeded previously reported national (17.1%) and single-centre clinic-based (6.0%) estimates (Mohamad et al., 2022), suggesting that prescribing practices were influenced by patient acuity, healthcare setting, and local clinical decision-making. During the pandemic, diagnostic uncertainty and the difficulty of distinguishing viral pneumonitis from bacterial co-infection increased reliance on empirical therapy, challenging pre-pandemic AMS approaches that were more dependent on structured diagnostic pathways and antimicrobial review processes (Elshenawy, 2025; Pierce & Stevens, 2021).

Consistent with broader evidence, antimicrobial prescribing in our cohort was associated with COVID-19 severity. Patients with severe or critical disease received more antibiotics, likely reflecting greater clinical complexity, prolonged hospitalisation, and the challenge of excluding secondary bacterial infections when microbiological confirmation was unavailable (Abu-Rub et al., 2021; Choi et al., 2023; Dominguez et al., 2023). Similarly, antifungal therapy was observed exclusively among critically ill patients, reflecting their increased vulnerability to invasive fungal infections during severe COVID-19 and intensive care exposure (Pruthi, 2022).

MDROs were detected in 8.4% of our study population, which was lower than global patient-level estimates (26.9–49.5%) (Langford et al., 2023). Gram-negative organisms predominated, with *S. maltophilia* accounting for nearly half of MDRO isolates. The prominence of *S. maltophilia* is consistent with reports from other geographical regions, although the reported prevalence rates vary substantially due to differences in patient populations, ICU exposure, antimicrobial pressure, infection control practices, and microbiological surveillance strategies (Baiou et al., 2021; Raad et al., 2023). These contextual differences likely explain the variation in MDRO prevalence reported during the pandemic rather than reflecting a uniform global trend.

A dip in DOT during April 2021 coincided with lower admissions. However, the disproportionately high DOT in February 2021 despite fewer cases, and the two-month lag between peak admissions in September and peak DOT in November, suggest delayed antimicrobial escalation during periods of sustained high disease burden. Although temporal DOT patterns were not fully aligned with admission rates in this study, they provide a low-cost metric to monitor antimicrobial demand, guide procurement and staffing, and support responsive antimicrobial stewardship and surge preparedness (Yarrington & Moehring, 2019).

The association between antimicrobial consumption and MDRO emergence should be interpreted considering potential time lags, as previous evidence demonstrates that resistance emergence following antibiotic exposure may unfold over the first six-month period (Poku et al., 2023). In this study, a potential lag was observed during the third wave, with antimicrobial consumption peaking in November 2021, approximately two months after peak COVID-19 admissions in September 2021, while MDRO rates remained elevated into late 2021. Future studies using longer time-series data and lag-based modelling approaches are needed to better quantify these temporal relationships.

Although seasonal respiratory viruses such as influenza may influence antimicrobial prescribing, influenza circulation in Malaysia remained substantially suppressed during 2020–2021 due to pandemic-related public health interventions, including movement restrictions and masking (Tang & Chin, 2021). Therefore, influenza co-circulation was unlikely to be a major contributor to the observed antimicrobial trends.

Our staged modelling identified severe/critical COVID-19 stage, comorbidities, elevated neutrophil counts, and elevated CRP as consistent predictors of antimicrobial use, whereas sociodemographic factors showed limited influence. These findings suggest that antimicrobial prescribing decisions were primarily guided by clinical severity and inflammatory markers**.** Similar observations have been reported internationally, where clinicians relied on severity indicators and inflammatory markers to support empirical antimicrobial decisions in the absence of definitive microbiological confirmation (Ming et al., 2021; Mohamad et al., 2022; Yang et al., 2024).

Variables included in the minimal model were selected based on prior literature. Dyslipidaemia was considered because of its potential association with COVID-19 severity; however, it was not retained in the final model due to insufficient information regarding disease severity and control. Although dyslipidaemia was associated with antimicrobial use in univariate analysis, its effect was attenuated after adjustment, suggesting that underlying clinical severity may be a stronger determinant of prescribing decisions (Bellia et al., 2021; Mohamad et al., 2022; Yang et al., 2024).

The impact of smoking on COVID-19 outcomes remains inconclusive, with studies reporting both immunomodulatory effects of nicotine and harmful effects of cigarette smoke (Grigg, 2022; Salehi et al., 2023). In our study, smoking was significantly associated with higher antimicrobial use. This association may reflect increased susceptibility to respiratory infections or more severe clinical presentation among smokers (Jiang et al., 2020). However, as prescribing decision-making was not directly examined, this finding should be interpreted as hypothesis-generating rather than indicative of clinician prescribing bias.

Pre-hospital steroid or immunosuppressant use was associated with a modestly higher rate of antimicrobial prescribing in our study. Patients with high-dose immunosuppression (e.g. corticosteroids ≥30 mg or biologics) received more antimicrobial therapy during COVID-19 hospitalisation despite lacking higher rates of confirmed secondary infection (9.3%) (Crook et al., 2023). While general immunosuppressant exposure may not worsen overall COVID-19 outcomes, prior high-dose glucocorticoid use (≥20 mg/day) has been linked to increased risks of hospitalisation and mortality in COVID-19 patients (Brodin et al., 2022). Consequently, clinicians likely adopt a proactive approach, maintaining a low threshold for empiric antimicrobial therapy in patients with prior high-dose steroid exposure.

In contrast, vaccination status was not associated with antimicrobial use as indicated in the extended model, despite evidence showing that vaccination in older populations reduces antibiotic prescribing (Jorgensen et al., 2024).

## Limitations

This study has several limitations. Reliance on clinical records may have introduced incomplete documentation, and unmeasured confounders – such as prior antimicrobial use and local resistance patterns – could have influenced prescribing and MDRO prevalence (Gearing et al., 2006). Despite training, use of standardised CRFs, and independent verification of a random 10% of records, a small degree of abstraction bias may remain due to variability in medical record documentation. Although confined to a single state, this multicentre study provided detailed state-wide insights, with COVID-19 admission trends mirroring national patterns (Tan et al., 2021).

This study analysed a stratified random sample of eligible admissions rather than the total volume of 19,116 patients. Because protocol constraints limited clinical data extraction to sampled individuals, direct comparisons between selected and non-selected admissions were not possible, potentially limiting sample representativeness. However, probability-based stratified sampling by state-level bed capacity was used to minimise selection bias, though residual risk relative to total volume remains a limitation.

Prescription appropriateness was not assessed, and pandemic restrictions limited data collection, underscoring the need for crisis-adapted protocols and trained research teams. Incomplete culture testing may have affected MDRO and secondary infection data. Only 119 of 167 antimicrobial recipients (71.3%) underwent culture testing. Because testing was performed based on clinical indication rather than universally, this potentially introduced selection bias. Patients tested were likely those with higher suspicion of bacterial infection or greater clinical severity. Therefore, the reported MDRO prevalence reflects only the tested subgroup and may overestimate or underestimate the true burden among all antimicrobial recipients, emphasising the need for comprehensive microbiological surveillance in future studies.

Pearson’s correlation was used to assess the association between monthly COVID-19 admissions and antimicrobial utilisation. However, as both variables are time-series data, this analysis did not account for autocorrelation or secular trends, potentially violating the independence assumption and inflating the observed correlation. Future stewardship research should utilise time-series analysis with autoregressive modelling to adjust for background prescribing trends and better guide antimicrobial policies during future pandemics.

While multiple imputation was performed using five imputations, our stability analysis demonstrated excellent replicability across random seeds, with CV <5% for the key imputed variables. This suggests that additional imputations would be unlikely to meaningfully alter our conclusions (Hippel, 2018; White et al., 2011). Nevertheless, the relatively higher variability observed for lymphocytes (CV = 29.8%) indicates that estimates involving this variable should be interpreted with caution.

## Conclusion

This multicentre study found that antimicrobial use and MDRO prevalence among hospitalised COVID-19 patients in Northern Malaysia appeared lower than those reported internationally, although incomplete culture testing and subgroup-specific denominators limit direct comparisons. Higher antimicrobial prescribing was consistently associated with severe or critical COVID-19 stage, selected comorbidities (DM, COPD, and CKD), longer hospital stays, and elevated inflammatory markers (neutrophil count and CRP) across both primary and extended models. When adjusting for extended clinical profiles, smoking history and pre-hospital steroid or immunosuppressant use were also significantly associated with increased antimicrobial exposure. At the population level, antimicrobial utilisation demonstrated distinct temporal patterns, including increased use early in the pandemic and a delayed peak following admission surges. These findings highlight the value of integrating clinical severity, inflammatory markers, and real-time antimicrobial surveillance to support outbreak-responsive antimicrobial stewardship.

## Supplementary Material

Supplemental Material - Appendices

## Data Availability

Data in this study belongs to the Perak State Health Department under the Malaysian Ministry of Health. Hence, it cannot be shared publicly. However, with a reasonable request, should any party require the data, they can send their request to the corresponding author with permission from the Director General of Health prior to being shared with any party.

## Abbreviations

**Abbreviations:** AMS: Antimicrobial stewardship; AUC: Area under the receiver operating characteristic curve; BMI: Body mass index; CKD: Chronic kidney disease; COPD: Chronic obstructive pulmonary disease; CRE: Carbapenem-resistant Enterobacterales; CI: Confidence interval; CRF: Case report form; CRP: C-reactive protein; CV: Coefficient of variation; DM: Diabetes mellitus; DOT: Days of therapy; ESBL: Extended spectrum beta-lactamase; GLMM: Generalized linear mixed model; HAI: Healthcare-associated infections; ICU: Intensive care unit; IQR: Interquartile range; IRR: Incidence rate ratio; MAR: Missing at random; MCAR: Missing completely at random; MDR: Multidrug-resistant; MDRO: Multidrug-resistant organisms; MRSA: Methicillin-resistant Staphylococcus aureus; OR: Odd ratio; RTK-Ag: Antigen rapid test kit; RT-PCR: Reverse transcription polymerase chain reaction; SD: Standard deviation; *spp.*: Species pluralis; UTI: Urinary tract infection

## Generative artificial intelligence (AI)

Artificial intelligence–based tools (ChatGPT and DeepSeek) were used solely for language editing and grammatical refinement during manuscript preparation; all scientific content, analysis, and interpretations were generated by the authors.
