# Supplementary material for: In-hospital antimicrobial use and multidrug-resistant infections among COVID-19 patients in Northern Malaysia (COVAM): a multicentre study
Source: J Pharm Policy Pract. 2026 Sep 28;19(1):2728836. doi: 10.1080/20523211.2026.2728836 (PMC13629810; doi:10.1080/20523211.2026.2728836)
Supplement: Supplementary file 1 [file JPPP_A_2728836_SM3890.docx]

# Appendix 1: Case Report Form

| **Section A: Sociodemographic data** | | | | | | |
| --- | --- | --- | --- | --- | --- | --- |
| Subject ID | \|____________\|___\|___\|___\|  *(eg. HRPB001; Alphabet based on hospital initial)* |  | Pregnant *(Only applicable to female)* | 🞎 Yes, \|___\|___\| gestational weeks  🞎 No | Nationality | 🞎 Malaysian  🞎 Non Malaysian |
| Age | *(In completed year)* |  | Smoking status | 🞎 Active smoker  🞎 Ex-smoker  🞎 Non-smoker  🞎 Unknown | Ethnicity  *(applicable for Malaysian ONLY)* | 🞎 Malay  🞎 Chinese  🞎 Indian  🞎 Others, _______________________ |
| Gender | 🞎 Male  🞎 Female |  |  |  |  |  |

| **Section B: Baseline Clinical Data** | | | | | | | | |
| --- | --- | --- | --- | --- | --- | --- | --- | --- |
| Admission  *(For current hospitalization)* | Date of admission:  \|___\|___\|-\|___\|___\|-\|___\|___\|___\|___\|  DD MM YYYY  Admission ward: 🞎 ICU 🞎 Non-ICU |  | COVID-19 swab test date | \|___\|___\|-\|___\|___\|-\|___\|___\|___\|___\|  DD MM YYYY  *(Including RT-PCR/rapid molecular/RTK-Ag)* | |  | Comorbid  *(Can mark more than one)* | 🞎 None  🞎 Diabetes Mellitus *(including type 1 and type 2)*  🞎 Hypertension  🞎 Dyslipidaemia  🞎 Heart conditions *(including Congenital Heart Disease)*  🞎 Asthma  🞎 Chronic obstruction pulmonary disease  🞎 Chronic Pulmonary Disease *(not Asthma)*  🞎 Chronic Kidney Disease *(including End Stage Renal Disease)*  🞎 Liver Disease  🞎 HIV  🞎 Neurologic condition *(including dementia)*  🞎 Cerebrovascular disease  🞎 Obesity (BMI >30 kg/m^2^)  🞎 Overweight (BMI 25 to 29 kg/m^2^)  🞎 Down syndrome  🞎 Cancer  🞎 Sickle cell disease or thalassemia  🞎 Substance use disorders  🞎 Immune deficiencies  🞎 Others, __________________________________________________________________________________________ |
|  |  |  | COVID-19 Day of illness | \|___\|___\| days of illness | |  |  |  |
| Ward transfer  *(If more than 3 ward transfers, please add supplemental sheet)* | Date of transfer:  \|___\|___\|-\|___\|___\|-\|___\|___\|___\|___\|  DD MM YYYY  Transferred to: 🞎 ICU 🞎 Non-ICU |  | COVID-19 clinical staging upon admission | 🞎 Category 1  🞎 Category 2  🞎 Category 3 | 🞎 Category 4  🞎 Category 5 |  |  |  |
|  |  |  | Worst COVID-19 clinical staging | 🞎 Category 1  🞎 Category 2  🞎 Category 3 | 🞎 Category 4  🞎 Category 5 |  |  |  |
|  | Date of transfer:  \|___\|___\|-\|___\|___\|-\|___\|___\|___\|___\|  DD MM YYYY  Transferred to: 🞎 ICU 🞎 Non-ICU |  |  |  |  |  |  |  |
|  |  |  | Highest Oxygen Requirement | 🞎 Nasal prong  🞎 Facemask  🞎 High flow mask  🞎 High flow nasal cannula  🞎 Non-invasive ventilation  🞎 Mechanical ventilation | |  |  |  |
|  | Date of transfer:  \|___\|___\|-\|___\|___\|-\|___\|___\|___\|___\|  DD MM YYYY  Transferred to: 🞎 ICU 🞎 Non-ICU |  |  |  |  |  |  |  |
| COVID-19 vaccination status upon admission | 🞎 Not taken any dose  🞎 Completed first dose  🞎 Completed second dose  🞎 Completed booster dose  🞎 Unknown |  | Corticosteroid or other immunosuppressive medications prior to disease onset | 🞎 Yes  🞎 No  🞎 Unknown | |  |  |  |
|  |  |  | Height | 🞎 \|___\|___\|___\|.\|___\| cm  🞎 Not available | |  |  |  |
|  |  |  | Weight | 🞎 \|___\|___\|___\|.\|___\| kg  🞎 Not available | |  |  |  |

| **Section C: COVID-19 treatment** *(From diagnosis of COVID-19 until discharge)* | | | | | |
| --- | --- | --- | --- | --- | --- |
| Steroid | 🞎 Yes, \|___\|___\|___\| days  🞎 No |  | Other COVID-19 treatment  *(From diagnosis of COVID-19 until discharge)* | 🞎 None  🞎 Favipiravir  🞎 Remdesivir  🞎 Hydroxychloroquine  🞎 Interferon β-1b | 🞎 Lopivanir/Ritonavir (Kaletra)  🞎 Ribavirin  🞎 Ivermectin  🞎 Intravenous Ascorbic Acid  🞎 Others,_________________________________ |
| Immunomodulator | 🞎 Not given  🞎 Tocilizumab  🞎 Baricitinib |  |  |  |  |

| **Section D: Laboratory Parameters** *(Take the nearest results* ***PRIOR*** *to the time point)* | | | | | | | | |
| --- | --- | --- | --- | --- | --- | --- | --- | --- |
| Date (dd/mm/yyyy) | |  |  |  |  |  |  |  |
| Antimicrobial status | | Upon admission  *(Current hospitalization)* | Upon initiation of antimicrobial  *(Includes data from previous hospitalization for COVID-19 infection)* | 🞎 Additional  🞎 Escalation  🞎 De-escalation | 🞎 Additional  🞎 Escalation  🞎 De-escalation | 🞎 Additional  🞎 Escalation  🞎 De-escalation | 🞎 Additional  🞎 Escalation  🞎 De-escalation | 🞎 Additional  🞎 Escalation  🞎 De-escalation |
| Full blood count | White blood cells (10^9^/L) |  |  |  |  |  |  |  |
|  | [Hemoglobin](about:blank) (g/dL) |  |  |  |  |  |  |  |
|  | Platelets (10^9^/L) |  |  |  |  |  |  |  |
|  | Neutrophil Count (10^9^/L) |  |  |  |  |  |  |  |
|  | Lymphocyte Count (10^9^/L) |  |  |  |  |  |  |  |
| Inflammatory markers | C-reactive Protein (CRP) |  |  |  |  |  |  |  |
|  | Procalcitonin (µg/L) |  |  |  |  |  |  |  |
| Liver Function test | Alanine transaminase (U/L) |  |  |  |  |  |  |  |
|  | Aspartate transaminase (U/L) |  |  |  |  |  |  |  |
|  | Alkaline phosphatase (U/L) |  |  |  |  |  |  |  |
|  | Albumin (g/L) |  |  |  |  |  |  |  |
|  | Total Bilirubin (µmol/L) |  |  |  |  |  |  |  |
| Renal Profile | Serum creatinine (µmol/L) |  |  |  |  |  |  |  |
|  | Creatinine clearance (mL/min) |  |  |  |  |  |  |  |

| **Section E: Antibiotic Prescription** *(please add the supplementary form if more than 5 entries)* | | | | | | | | | |
| --- | --- | --- | --- | --- | --- | --- | --- | --- | --- |
| Was antibiotic prescribed during COVID-19 infection? | | 🞎 Yes *(Complete Section E)*  🞎 No *(Proceed to Section F)* | |  | COVID-19 clinical staging upon initiation of antibiotic | 🞎 Category 1  🞎 Category 2  🞎 Category 3 | | 🞎 Category 4  🞎 Category 5 | |
|  | | | | | | | | | |
| Antibiotic Name | Date Started | | Date Stopped | | Diagnosis | | Location when prescribed | | Indication |
| 1. | \|___\|___\|-\|___\|___\|-\|___\|___\|___\|___\|  DD MM YYYY | | \|___\|___\|-\|___\|___\|-\|___\|___\|___\|___\|  DD MM YYYY | | 🞎 Pneumonia  🞎 Bacteremia  🞎 Urinary tract infection  🞎 Central nervous system infection  🞎 Skin and soft tissue infection  🞎 Others, ______________________ | | 🞎 ICU  🞎 Non-ICU | | 🞎 Empirical  🞎 Definitive  🞎 Prophylaxis |
| 2. | \|___\|___\|-\|___\|___\|-\|___\|___\|___\|___\|  DD MM YYYY | | \|___\|___\|-\|___\|___\|-\|___\|___\|___\|___\|  DD MM YYYY | | 🞎 Pneumonia  🞎 Bacteremia  🞎 Urinary tract infection  🞎 Central nervous system infection  🞎 Skin and soft tissue infection  🞎 Others, ______________________ | | 🞎 ICU  🞎 Non-ICU | | 🞎 Empirical  🞎 Definitive  🞎 Prophylaxis |
| 3. | \|___\|___\|-\|___\|___\|-\|___\|___\|___\|___\|  DD MM YYYY | | \|___\|___\|-\|___\|___\|-\|___\|___\|___\|___\|  DD MM YYYY | | 🞎 Pneumonia  🞎 Bacteremia  🞎 Urinary tract infection  🞎 Central nervous system infection  🞎 Skin and soft tissue infection  🞎 Others, ______________________ | | 🞎 ICU  🞎 Non-ICU | | 🞎 Empirical  🞎 Definitive  🞎 Prophylaxis |
| 4. | \|___\|___\|-\|___\|___\|-\|___\|___\|___\|___\|  DD MM YYYY | | \|___\|___\|-\|___\|___\|-\|___\|___\|___\|___\|  DD MM YYYY | | 🞎 Pneumonia  🞎 Bacteremia  🞎 Urinary tract infection  🞎 Central nervous system infection  🞎 Skin and soft tissue infection  🞎 Others, ______________________ | | 🞎 ICU  🞎 Non-ICU | | 🞎 Empirical  🞎 Definitive  🞎 Prophylaxis |
| 5. | \|___\|___\|-\|___\|___\|-\|___\|___\|___\|___\|  DD MM YYYY | | \|___\|___\|-\|___\|___\|-\|___\|___\|___\|___\|  DD MM YYYY | | 🞎 Pneumonia  🞎 Bacteremia  🞎 Urinary tract infection  🞎 Central nervous system infection  🞎 Skin and soft tissue infection  🞎 Others, ______________________ | | 🞎 ICU  🞎 Non-ICU | | 🞎 Empirical  🞎 Definitive  🞎 Prophylaxis |

| **Section F: Antifungal Prescription** *(please add the supplementary form if more than 5 entries)* | | | | | | | | | |
| --- | --- | --- | --- | --- | --- | --- | --- | --- | --- |
| Was antifungal prescribed during COVID-19 infection? | | 🞎 Yes *(Complete Section F)*  🞎 No *(Proceed to Section G)* | |  | COVID-19 clinical staging upon initiation of antifungal | 🞎 Category 1  🞎 Category 2  🞎 Category 3 | | 🞎 Category 4  🞎 Category 5 | |
|  | | | | | | | | | |
| Antifungal Name | Date Started | | Date Stopped | | Diagnosis | | Location when prescribed | | Indication |
| 1. | \|___\|___\|-\|___\|___\|-\|___\|___\|___\|___\|  DD MM YYYY | | \|___\|___\|-\|___\|___\|-\|___\|___\|___\|___\|  DD MM YYYY | | 🞎 Pneumonia  🞎 Bacteremia  🞎 Urinary tract infection  🞎 Central nervous system infection  🞎 Skin and soft tissue infection  🞎 Others, ______________________ | | 🞎 ICU  🞎 Non-ICU | | 🞎 Empirical  🞎 Pre-emptive  🞎 Targeted |
| 2. | \|___\|___\|-\|___\|___\|-\|___\|___\|___\|___\|  DD MM YYYY | | \|___\|___\|-\|___\|___\|-\|___\|___\|___\|___\|  DD MM YYYY | | 🞎 Pneumonia  🞎 Bacteremia  🞎 Urinary tract infection  🞎 Central nervous system infection  🞎 Skin and soft tissue infection  🞎 Others, ______________________ | | 🞎 ICU  🞎 Non-ICU | | 🞎 Empirical  🞎 Pre-emptive  🞎 Targeted |
| 3. | \|___\|___\|-\|___\|___\|-\|___\|___\|___\|___\|  DD MM YYYY | | \|___\|___\|-\|___\|___\|-\|___\|___\|___\|___\|  DD MM YYYY | | 🞎 Pneumonia  🞎 Bacteremia  🞎 Urinary tract infection  🞎 Central nervous system infection  🞎 Skin and soft tissue infection  🞎 Others, ______________________ | | 🞎 ICU  🞎 Non-ICU | | 🞎 Empirical  🞎 Pre-emptive  🞎 Targeted |
| 4. | \|___\|___\|-\|___\|___\|-\|___\|___\|___\|___\|  DD MM YYYY | | \|___\|___\|-\|___\|___\|-\|___\|___\|___\|___\|  DD MM YYYY | | 🞎 Pneumonia  🞎 Bacteremia  🞎 Urinary tract infection  🞎 Central nervous system infection  🞎 Skin and soft tissue infection  🞎 Others, ______________________ | | 🞎 ICU  🞎 Non-ICU | | 🞎 Empirical  🞎 Pre-emptive  🞎 Targeted |
| 5. | \|___\|___\|-\|___\|___\|-\|___\|___\|___\|___\|  DD MM YYYY | | \|___\|___\|-\|___\|___\|-\|___\|___\|___\|___\|  DD MM YYYY | | 🞎 Pneumonia  🞎 Bacteremia  🞎 Urinary tract infection  🞎 Central nervous system infection  🞎 Skin and soft tissue infection  🞎 Others, ______________________ | | 🞎 ICU  🞎 Non-ICU | | 🞎 Empirical  🞎 Pre-emptive  🞎 Targeted |

| **Section G: Culture and Sensitivity Results** *(please add the supplementary form if more than 4 entries)* | | | | |
| --- | --- | --- | --- | --- |
| Culture and Sensitivity done during COVID-19 infection? | | 🞎 Yes *(Complete Section G)*  🞎 No *(Proceed to Section H)* | | |
|  | | | | |
| Sampling Source | Sampling Date | Result Date | Culture result | MRO present |
| 1. 🞎 Blood  🞎 Sputum  🞎 Bronchoalveolar lavage  🞎 Urine  🞎 Others, _____________________ | \|___\|___\|-\|___\|___\|-\|___\|___\|___\|___\|  DD MM YYYY | \|___\|___\|-\|___\|___\|-\|___\|___\|___\|___\|  DD MM YYYY | 🞎 No growth  🞎 Positive, specify:  ____________________________________________________________________________________________________________________________________ | 🞎 Yes  🞎 No |
| 2. 🞎 Blood  🞎 Sputum  🞎 Bronchoalveolar lavage  🞎 Urine  🞎 Others, _____________________ | \|___\|___\|-\|___\|___\|-\|___\|___\|___\|___\|  DD MM YYYY | \|___\|___\|-\|___\|___\|-\|___\|___\|___\|___\|  DD MM YYYY | 🞎 No growth  🞎 Positive, specify:  ____________________________________________________________________________________________________________________________________ | 🞎 Yes  🞎 No |
| 3. 🞎 Blood  🞎 Sputum  🞎 Bronchoalveolar lavage  🞎 Urine  🞎 Others, _____________________ | \|___\|___\|-\|___\|___\|-\|___\|___\|___\|___\|  DD MM YYYY | \|___\|___\|-\|___\|___\|-\|___\|___\|___\|___\|  DD MM YYYY | 🞎 No growth  🞎 Positive, specify:  ____________________________________________________________________________________________________________________________________ | 🞎 Yes  🞎 No |
| 4. 🞎 Blood  🞎 Sputum  🞎 Bronchoalveolar lavage  🞎 Urine  🞎 Others, _____________________ | \|___\|___\|-\|___\|___\|-\|___\|___\|___\|___\|  DD MM YYYY | \|___\|___\|-\|___\|___\|-\|___\|___\|___\|___\|  DD MM YYYY | 🞎 No growth  🞎 Positive, specify:  ____________________________________________________________________________________________________________________________________ | 🞎 Yes  🞎 No |
| 5. 🞎 Blood  🞎 Sputum  🞎 Bronchoalveolar lavage  🞎 Urine  🞎 Others, _____________________ | \|___\|___\|-\|___\|___\|-\|___\|___\|___\|___\|  DD MM YYYY | \|___\|___\|-\|___\|___\|-\|___\|___\|___\|___\|  DD MM YYYY | 🞎 No growth  🞎 Positive, specify:  ____________________________________________________________________________________________________________________________________ | 🞎 Yes  🞎 No |

**Section H: COVID-19 Complication** *(May mark more than 1)*

🞎 None

🞎 Organizing pneumonia

🞎 Pulmonary embolism

🞎 Acute kidney injury (including acute on chronic kidney disease)

🞎 Others, ___________________________________________________________________________________________

**Section I: Clinical Outcome** *(Please mark ONE only)*

🞎 Alive and discharge well

Date: |___|___|-|___|___|-|___|___|___|___|

DD	MM	YYYY

🞎 Transfer out to other hospital facilities

Date: |___|___|-|___|___|-|___|___|___|___|

DD	MM	YYYY

🞎 AOR Discharge

Date: |___|___|-|___|___|-|___|___|___|___|

DD	MM	YYYY

🞎 Death

Date: |___|___|-|___|___|-|___|___|___|___|

DD	MM	YYYY

Cause of death: ___________________________________

| **Data Collector Information** | |
| --- | --- |
| Signature and stamp |  |
| Date of completion | \|___\|___\|-\|___\|___\|-\|___\|___\|___\|___\|  DD MM YYYY |

# Appendix 2: Descriptive analysis of imputed data

| Variable | Original Mean (SD) | Imputed Mean (SD)  5 sets | Mean Difference | t-statistic | p-value | Statistical Significance (α=0.05) |
| --- | --- | --- | --- | --- | --- | --- |
| CRP | 77.47 (92.27) | 74.91 (101.62) | 2.56 | 1.01 | 0.312 | Not Significant |
| Lymp | 1.64 (4.61) | 1.93 (8.80) | -0.29 | -1.52 | 0.129 | Not Significant |
| Neu | 7.95 (11.46) | 7.94 (14.02) | 0.01 | 0.02 | 0.984 | Not Significant |

Abbreviations: CRP: C-reactive protein; Lymp: Lymphocytes; Neu: Neutrophil

# Appendix 3: Variability of biomarkers across random seed initializations

| Variable | Mean (Across 5 random seeds) | SD (Across Seeds) | CV (%) |
| --- | --- | --- | --- |
| CRP | 54.60 | 2.45 | 4.49 |
| Lym | 2.69 | 0.80 | 29.82 |
| Neu | 5.34 | 0.11 | 2.11 |

Abbreviations: CRP: C-reactive protein; Lymp: Lymphocytes; Neu: Neutrophil, SD = Standard Deviation; CV = Coefficient of Variation (CV = SD/Mean × 100%).

Five random seeds, mean, SD, and CV were generated with RStudio 2025.09.2+418 "Cucumberleaf Sunflower" (12f6d5e2, 2025-10-20) for Windows, with Quarto 1.7.32: 2024, 12345, 24680, 13579, 54321.

# Appendix 4: Number of eligible patients and their allocation by hospital

| Study site | Type of hospital | ICU | AMS team | Eligible cases for sampling | No. of Bed | Proportionate sampling based on bed capacity | Withdrawn cases | Cases included for analysis |
| --- | --- | --- | --- | --- | --- | --- | --- | --- |
| Hospital Raja Permaisuri Bainun | State hospital | Yes | Yes | 5,170 | 990 | 153 | 2 | 151 |
| Hospital Taiping | Specialist Hospital | Yes | Yes | 3,906 | 608 | 94 | 1 | 93 |
| Hospital Teluk Intan | Specialist Hospital | Yes | Yes | 2,860 | 548 | 85 | 0 | 85 |
| Hospital Seri Manjung | Specialist Hospital | Yes | Yes | 2,167 | 305 | 47 | 0 | 47 |
| Hospital Kuala Kangsar | Non-Specialist Hospital | No | Yes | 556 | 136 | 21 | 0 | 21 |
| Hospital Slim River | Minor Specialist Hospital | Yes | Yes | 1,270 | 115 | 18 | 0 | 18 |
| Hospital Parit Buntar | Non-Specialist Hospital | No | Yes | 683 | 100 | 16 | 0 | 16 |
| Hospital Batu Gajah | Non-Specialist Hospital | No | Yes | 1,081 | 100 | 16 | 0 | 16 |
| Hospital Tapah | Non-Specialist Hospital | No | Yes | 135 | 100 | 16 | 0 | 16 |
| Hospital Sungai Siput | Non-Specialist Hospital | No | Yes | 1,288 | 93 | 14 | 1 | 13 |
|  | |  | Total | 19,116 | 3095 | 480 | 4 | 476 |

Abbreviation. AMS: Antimicrobial Stewardship programs, ICU: Intensive care uni.

Hospital types were defined as follows: non-specialist hospitals provide basic inpatient and emergency services without resident specialists; specialist hospitals have resident specialists providing specialist clinical services; minor specialist hospitals provide limited specialist services; and state hospitals are main state referral hospitals offering comprehensive specialist and subspecialty services.

# Appendix 5: Clinical characteristics of hospitalized COVID-19 patients

| **Variables** |  | Total, n (%)  (n=476) | Received antimicrobial  (n=167) | p-value^a^ |
| --- | --- | --- | --- | --- |
| COVID-19 clinical stage |  |  |  |  |
| Upon admission | Mild to moderate | 326 (68.5) | 68 (20.9) | <0.001 |
|  | Severe to critically ill | 150 (31.5) | 99 (66.0) |  |
| Worst during hospitalization | Mild to moderate | 291 (61.1) | 49 (16.8) | <0.001 |
|  | Severe to critically ill | 185 (38.9) | 118 (63.8) |  |
| Laboratory profile (out-of-normal range) | |  |  |  |
| White blood cells count | Admission (n=374) | 72 (19.3) | 44 (61.1) |  |
|  | At initiation (n=158) | 60 (38.0) | 60 (100.0) |  |
| Platelet count | Admission (n=375) | 78 (20.8) | 36 (46.2) |  |
|  | At initiation (n=158) | 40 (25.3) | 40 (100.0) |  |
| Neutrophil count | Admission (n=322) | 98 (30.4) | 62 (63.3) |  |
|  | At initiation (n=151) | 76 (50.3) | 76 (100.0) |  |
| Lymphocyte count | Admission (n=353) | 121 (34.3) | 69 (57.0) |  |
|  | At initiation (n=157) | 66 (42.0) | 66 (100.0) |  |
| C-reactive protein level | Admission (n=316) | 256 (81.0) | 110 (43.0) |  |
|  | At initiation (n=453) | 448 (98.9) | 139 (31.0) |  |
| Alanine transaminase | Admission (n=343) | 281 (81.9) | 97 (34.5) |  |
|  | At initiation (n=120) | 89 (74.2) | 89 (100.0) |  |
| Aspartate transaminase | Admission (n=312) | 136 (43.6) | 73 (53.7) |  |
|  | At initiation (n=113) | 71 (62.8) | 71 (100.0) |  |
| Albumin | Admission (n=342) | 163 (47.7) | 86 (52.8) |  |
|  | At initiation (n=120) | 86 (71.7) | 86 (100.0) |  |
| Total bilirubin | Admission (n=342) | 54 (15.8) | 24 (44.4) |  |
|  | At initiation (n=117) | 17 (14.5) | 17 (100.0) |  |
| Highest oxygen requirement during hospitalization | Not required | 276 (58.0) | 44 (15.9) | <0.001 |
|  | Oxygen supplement^b^ | 139 (29.2) | 67 (48.2) |  |
|  | Non-invasive & invasive ventilation^c^ | 61 (12.8) | 56 (91.8) |  |

"At initiation" refers to the time when antimicrobial treatment was started. Percentages shown as 100% indicate that all patients with abnormal laboratory values were, by definition, receiving antimicrobial therapy. For example, among the 158 patients tested for white blood cell count at initiation, 60 (38.0%) had deranged values, and all 60 (100%) received antimicrobials.

^a^Analyzed with the χ^2^-test, ^b^Nasal prong, facemask, and high-flow mask, ^c^High-flow nasal cannula, non-invasive ventilation, and invasive mechanical ventilation.

Normal-range laboratory parameters: White blood cells (4.0 – 11.0x10^9^/L), platelet (150 – 450x10^9^/L), Neutrophil (2.0 – 7.0x10^9^/L), Lymphocyte (1.0 – 3.0x10^9^/L), C-reactive protein (0 – 5 mg/L), Alanine transaminase (0 – 33 U/L), Aspartate transaminase (0 – 32 U/L), Albumin (35 – 52 g/L), Total bilirubin (1 – 17.1 µmol/L)

# Appendix 6: Classification of Antimicrobial Prescribing by COVID-19 Severity

| Class | Antimicrobial | Mild / Moderate  n = 200, n (%) | Severe / critical  n = 181, n (%) | *p*-value |
| --- | --- | --- | --- | --- |
| Beta-lactam inhibitor combinations | Amoxicillin/Clavulanate | 77 (58.8) | 54 (41.2) | <0.001 |
|  | Ampicillin/Sulbactam | 1 (5.3) | 18 (94.7) |  |
|  | Cefoperazone/Sulbactam | 2 (100.0) | 0 (0) |  |
|  | Piperacilin/Tazobactam | 5 (9.3) | 49 (90.7) |  |
| Penicillins | Ampicillin | 0 (0) | 1 (100.0) | 0.475 |
|  | Cloxacillin | 1 (50.0) | 1 (50.0) |  |
| Macrolides | Azithromycin | 45 (59.2) | 31 (40.8) | <0.001 |
| Cephalosporins | Cefazolin (1st) | 4 (100.0) | 0 (0) | 0.002 |
|  | Cephalexin (1st) | 0 (0) | 1 (100.0) |  |
|  | Cefuroxime (2nd) | 4 (22.2) | 14 (77.8) |  |
|  | Ceftazidime (3rd) | 10 (90.9) | 1 (9.1) |  |
|  | Ceftriaxone (3rd) | 8 (50.0) | 8 (50.0) |  |
|  | Cefepime (4th) | 7 (100) | 0 (0.0) |  |
| Lincosamide | Clindamycin | 1 (100) | 0 (0.0) | 1.000 |
| Carbapenems | Imipenem | 1 (100) | 0 (0.0) | <0.001 |
|  | Meropenem | 23 (95.8) | 1 (4.2) |  |
| Fluoroquinolones | Levofloxacin | 3 (100.0) | 0 (0.0) | 0.250 |
| Nitroimidazoles | Metronidazole | 0 (0.0) | 2 (100) | 0.217 |
| Glycopeptides | Vancomycin | 5 (100.0) | 0 (0) | 0.065 |
| Sulfonamides | Sulfamethoxazole/trimethoprim | 2 (100.0) | 0 (0) | 0.500 |
| Polymyxins | Colistin | 1 (100.0) | 0 (0) | 1.000 |
| Echinocandin | Anidulafungin | 0 (0.0) | 3 (100.0) | 1.000 |
|  | Caspofungin | 0 (0.0) | 1 (100.0) |  |
|  | Micafungin | 0 (0.0) | 5 (100.0) |  |
| Azole | Fluconazole | 0 (0.0) | 6 (100.0) | 1.000 |

Total N = 381 encounters of antimicrobial receipt.

*p*-values were calculated using a two-sided Fisher's exact test to compare the proportion of patients with mild/moderate versus critical/severe infections for each antimicrobial.

# Appendix 7: Factors Associated with number of antimicrobial prescriptions: Comparison of Minimal, Primary, and Extended Multivariable Regression Models

|  | | Minimal Model | | Primary Model | | Extended Model | |
| --- | --- | --- | --- | --- | --- | --- | --- |
| Variables | | IRR | 95% CI | IRR | 95% CI | IRR | 95% CI |
|  | Age | **1.001** | **1.000 - 1.002** | 0.999 | 0.997 - 1.001 | 0.999 | 0.997 - 1.001 |
|  | Gender (Male vs. Female) | **1.057** | **1.031 - 1.083** | 1.050 | 0.996 - 1.107 | **1.061** | **1.002 - 1.123** |
|  | COVID-19 Stage (Critical/severe vs. mild/moderate) | **3.515** | **3.420 - 3.612** | **1.769** | **1.646 - 1.900** | **1.763** | **1.641 - 1.895** |
|  | Diabetes Mellitus (Yes vs. No) | **1.139** | **1.107 - 1.172** | **1.369** | **1.279 - 1.466** | **1.384** | **1.292 - 1.483** |
|  | Hypertension (Yes vs. No) | **1.114** | **1.082 - 1.146** | **0.932** | **0.874 - 0.994** | **0.919** | **0.861 - 0.980** |
|  | COPD (Yes vs. No) | **1.302** | **1.192 - 1.423** | **1.254** | **1.023 - 1.538** | **1.262** | **1.022 - 1.558** |
|  | CKD (Yes vs. No) | **1.526** | **1.465 - 1.589** | **1.183** | **1.087 - 1.288** | **1.233** | **1.124 - 1.353** |
|  | Dyslipidaemia (Yes vs. No) |  |  | 1.058 | 0.987 - 1.134 | 1.069 | 0.996 - 1.147 |
|  | Hospital Stay (number of days) |  |  | **1.053** | **1.050 - 1.055** | **1.050** | **1.048 - 1.053** |
|  | Neutrophil Count |  |  | **1.008** | **1.007 - 1.010** | **1.008** | **1.007 - 1.010** |
|  | Lymphocyte Count |  |  | **0.976** | **0.958 - 0.995** | **0.975** | **0.956 - 0.995** |
|  | CRP |  |  | **1.001** | **1.0005 - 1.001** | **1.001** | **1.0005 - 1.001** |
|  | Smoking Status (Yes vs. no/unknown) |  |  |  |  | **1.102** | **1.019 - 1.193** |
|  | Pre-hospital steroid/immunosuppressant use (Yes vs. No) |  |  |  |  | **1.100** | **1.013 - 1.194** |
|  | COVID-19 Vaccination (1 Dose vs. 0) |  |  |  |  | 1.080 | 0.990 - 1.177 |
|  | COVID-19 Vaccination (2+ Doses vs. 0) |  |  |  |  | 1.026 | 0.950 - 1.108 |

Abbreviations: COPD, Chronic obstructive pulmonary disease; CKD, Chronic kidney disease; CRP: C-reactive protein; IRR: incidence rate ratios; 95% CI: 95% Confidence interval.
